# Seed Survival in Silage: Reviewing 90 Years of Research

**DOI:** 10.3390/plants14030351

**Published:** 2025-01-24

**Authors:** Juliane Hahn, Jürgen Müller, Monika Heiermann

**Affiliations:** 1 Department Technology Assessment, Leibniz Institute for Agricultural Engineering and Bioeconomy (ATB), 14469 Potsdam, Germany; mheiermann@atb-potsdam.de; 2 Crop Health, Faculty of Agricultural and Environmental Sciences, University of Rostock, 18051 Rostock, Germany; 3 Grassland and Forage Sciences, Faculty of Agricultural and Environmental Sciences, University of Rostock, 18051 Rostock, Germany; juergen.mueller3@uni-rostock.de

**Keywords:** biomass, circular bioeconomy, ensiling conditions, feedstock, hardseededness, physical dormancy, seed viability, sustainability, weed management

## Abstract

The preservation of biomasses through ensiling has a long history, and its sustainability has many aspects. One that is rarely considered is that the seeds of a wide variety of plants can enter the ensiling process with the plant biomass. This concise review provides an overview of the probability of seed survival in various types of silage since the 1930s. All data extracted from the reviewed studies are made available in a repository. The key finding from the 90 years of research is that ensiling can reduce the viability of plant seeds, but the seeds of some plant species can survive ensiling. Thus, silage production is both (1) a potential tool to ensure the sustainable, i.e., weed-free, use of plant biomass in agricultural production, including animal production chains, and (2) a potential gateway for weed spread, especially with regard to the closed material loops in circular bioeconomy approaches. The search for seed-borne factors and ensiling conditions that promote seed survival or killing is still ongoing and should be the subject of future research.

## 1. Seeds and Silage Sustainability

In temperate climate zones worldwide, ensiling is the most common form of forage preservation alongside drying [1,2]. Ensiling is based on the anaerobic fermentation of low-molecular carbohydrates in the feedstock with the formation of pH-lowering fermentation acids, mainly lactic acid [3]. While the fundamental biochemical principles of ensiling have not changed in the last century, significant progress has been made in the production technology of ensiling [4]. This progress has contributed to a reduction in energy losses and emissions [5] and, thus, to the greater sustainability of the ensiling process.

However, far less attention has been paid to aspects of the integration of ensiling into operational biomass flows and their effects on the sustainability of the entire biomass cycle. This extends from the field to utilization in animal husbandry (or the biorefinery) and to the utilization of manure (or digestate) [6]. One such aspect of silage biomass utilization barely considered so far is the targeted use of ensiling to reduce weed seed loads. Seeds that are killed during the ensiling process can neither cause problems in contaminated feed residues via the manure path on manure-fertilized fields, nor can they be stimulated to germinate by the gastrointestinal passage when feeding silage to livestock [7]. Remarkably, studies on this free, weed-reducing on-farm service of ensiling date back to the 1930s—a time when synthetic chemical herbicides were not yet widespread and integrated weed control was not an alternative [8,9].

Since the first study published in 1934, the issue of seed survival or killing during ensiling has rarely been addressed. Recently, however, a number of authors from different countries have studied this issue at the interface of seed biology and biomass process engineering [10,11,12]. Despite different agronomic backgrounds, this renewed interest is due to the fact that herbicide-based weed control methods practiced since the 1960s are reaching their limits, as evidenced by herbicide resistances [13] or soil and groundwater contamination with herbicide residues [14], for example. New, more sustainable weed control systems must, therefore, be developed [15]. In addition, the increase in invasive weed species [16] poses new challenges, as does the rise of circular bioeconomy approaches, in which ensiled biomass is of growing importance for biorefineries, and residual materials must be managed sustainably [17]. In the optimally closed biomass cycles [18], surviving and spreading weed seeds would have a negative impact on sustainability.

Studies on the survival of weed seeds in the fermentation process of ensiling are quite laborious, which is because in addition to the design of the ensiling conditions with their microbiological uncertainties, a number of experimental obstacles in handling the seeds have to be overcome, e.g., good exposure in the substrate, safe recovery and salvage, methodology for determining survival rate and germination capacity. Therefore, it seems important to present the findings gained so far in a condensed form and to evaluate them across cases in order to enable future experimenters to focus their research approaches specifically on the gaps in knowledge and to avoid redundancies or even inadequacies in the experimental design, documentation, and interpretation.

Against this background, the present review on seed survival in silages aimed to collect and analyze available data and to identify similarities and differences depending on the botanical classification of the plants, the physiological characteristics of the seeds, and the ensiling conditions. This was intended to provide a basis for giving practitioners an indication of which species are likely to survive ensiling or not, creating a database for decision makers, identifying potentially important aspects of seed biology, and encouraging further research in this field.

## 2. Literature Search, Data Sets, and Data Analyses

### 2.1. Literature Search

The literature search for this review was conducted from 2020 to 2024 via the platforms Scopus (https://www.scopus.com), Web of Science (https://www.webofscience.com), and Google Scholar (https://scholar.google.de/). The following keywords were searched for: seed, seeds, silage, ensiling, silage, survival, probability of survival, viability, and germination. All available publications in English, German, and Danish from the earliest possible date to the present were included.

### 2.2. Data Sets and Data Availability

The first data set on seed survival in silage was published in 1934 in William Shevkenek’s master’s thesis. Since then, a total of 23 publications have documented research on this topic (Table 1). Of these 23 publications, 16 were articles in peer-reviewed journals, 3 were conference papers, 3 were university publications, and 1 was a gray report. For this review, data on seed survival before and after different ensiling treatments could be extracted from 21 of the 23 available studies. All extracted values on seed survival and the respective ensiling treatments (see Section 2.2.1 and Section 2.2.2) were compiled and uploaded to the online repository ZENODO (https://zenodo.org/); see the Data Availability Statement. 

#### 2.2.1. Data on Seed Survival

Since 1934, the survival of 113 plant species in silage has been studied. The species originate from 22 different families: 38 species are monocotyledonous and 75 dicotyledonous. The highest number of species in one study, namely 26, was investigated by Woodward in 1940 [19]. A total of 15 studies compared seeds from different species [7,8,9,10,11,12,19,20,21,22,23,24,25,26,27], while the remaining studies focused on a single plant species (Table 1). 

On average (median), four replicates were examined, but many studies tested only one replicate. The number of seeds per replicate ranged from 15 [28] to 1000 [8] (Table 1).

Regarding seed viability, most studies tested seed germination (in pots, on filter paper, or on agar plates), and many of them additionally reported metabolic activity (by staining with 2,3,5-triphenyltetrazolium chloride, TTC). Occasionally, tests for physical integrity were also carried out (crush test with forceps). In some cases, however, it remained unclear which response parameter was measured to determine viability [9,25,29]. Therefore, the measures “survival”, “survival probability”, “viability”, and “germination” were considered as a viability response for data extraction.

From each study, seed viability data per species were extracted from tables, figures, or text. If more than one replicate was available, means or medians were extracted. If both germination and viability were provided, viability data were selected, and it was noted (in the repository) that germination data were available. Sample sizes and measures of variance such as standard deviations from the mean were not extracted. This approach yielded a total of 610 seed viability values from the available studies. For the comparison of the studies, the viability responses were normalized to those of the untreated controls. For this purpose, the viability response of the control was set to 100% and the responses of the treatments were converted in proportion to this. In cases where no viability data were provided for the controls, a viability of 100% was assumed.

In the repository, the following parameters and variables for seed survival were compiled for each study: species name, seed lot characteristics (if provided), plant family, plant class, potential to form physically dormant (hardseeded seeds), seed viability response, type of viability response, normalized viability response, percentage of loss of viability, survival (YES/NO), notes. The scientific names and family affiliations of plant species have been updated to the latest accepted names according to the Global Biodiversity Information Facility (GBIF) [30]. Therefore, the species name may differ from the name used in the original publications. Only one accepted name has been used for each species in this review.

**Table 1:** Chronological overview of studies examining seed survival in silages. From each study, information was extracted on the number of plant species tested in silage, the number of replicates, the response parameter measured (germination (g) and/or viability (v)), the substrate ensiled, the ensiling duration(s), any ensiling modifications, the type of silo in lab- or full-scale, and exposure of the seeds to further treatments (animal digestive tract, anaerobic digestion in biogas reactors, and storage in manure or similar).

Study	Year	Study Author(s)	Plant Species Number	Replicate Number (Seeds per Replicate)	Seed Response	Substrate(s) Ensiled	Ensiling Duration(s) [days] ^2^	Ensiling Modifications	Silo Scale			Further Treatments			Reference
									Lab	Full		Animal	Biogas Reactor	Storage	
A	1934	Shevkenek	6	1 (100–1000)	g	n.r.	105	-	-	n.r.		-	-	manure	[8]
B	1937	Tildesley	19	n.a.	v	n.a.	21	-	lab silo	-		-	-	-	[9]
C	1940	Woodward	26	n.r.	g	alfalfa; maize; alfalfa + grass	7–149	molasses; DM	-	stack		-	-	-	[19]
D	1941	Zahnley and Fitch	n.a.	n.a.	n.a.	maize; sorghum	n.a.	-	-	bunker		-	-	-	[31]
E	1959	Anonymous	14	1 (200–800)	g	sugar beet	60	-	-	bin		-	-	-	[20]
F	1991	Blackshaw and Rode	12	2 (150)	g + v	barley	56	-	-	bunker		bovine rumen	-	-	[7]
G	2000	Mayer et al.	6	1–10 (50)	g	grass	90, 120, 270, 360	-	-	bale		bovine rumen	-	manure, slurry	[21]
H	2002	Overud	1	4 (15–18)	g + v	clover + grass	14, 28, 42, 56, 61, 70, 100, 141	DM	glass jar	bale		-	-	-	[28]
I	2006	van Eekeren et al.	1	1 (n.r.)	v	grass	14, 28, 42, 56	DM	pot	-		-	-	-	[29]
J	2011	James et al.	1	5 (200)	g	grass; maize	90	-	-	stack; bale		-	-	effluent pond	[32]
K	2012	Lück	7	3 (100)	g + v	maize; rye	103, 155	-	glass jar	-		-	batch reactor	-	[22]
L	2012	Stanton et al.	14	4 (50)	g + v	cereals	90	-	plastic bag	-		bovine rumen	-	-	[23]
M	2012	Westerman, Hildebrandt et al.	17	3–10 (50–200)	g + v	maize; rye	135, 207	-	glass jar	-		-	batch reactor	-	[24]
N	2014	Aper et al.	1	4 (120)	g + v	maize	28, 49, 112	-	-	stack		bovine rumen	-	manure	[13]
O	2015	Koarai et al.	7	n.a.	v	rice	90, 180	-	n.a.	n.a.		-	-	-	[25]
P	2015	Trolove and Dowsett	1	n.a. (50)	g + v	n.a.	1, 2, 3, 4, 5, 6, 7	-	-	stack		-	-	-	[33]
Q	2016	Simard and Lambert- Beaudet	7	5 (100)	g + v	alfalfa; maize	30, 90, 180	-	lab silo	-		-	-	-	[11]
R	2016	Weller et al.	1	8 (25)	g + v	grass	7, 14, 35, 42	lactic acid bacteria	vacuum bag	-		-	-	-	[34]
S	2017	Piltz et al.	11	7 (50)	g + v	barley	90	-	plastic bag	-		bovine rumen	-	-	[10]
T	2021	Hahn et al.	10	2–9 (100–300)	g + v	maize; maize + wildflowers	237 or 281	soil suspension	glass jar	-		-	-	-	[12]
U	2021	Piltz et al.	16	3 (50)	g + v	alfalfa; clover + grass; sorghum; alfalfa chaff; cotton wool	14–147	organic acids; DM	plastic bag	-		bovine rumen	-	-	[26]
V	2022	Asaduzzaman et al.	2	3 (50)	g + v	alfalfa	90	-	plastic bag	-		bovine rumen	-	-	[27]
W	2023	Asaduzzaman et al.	1	3 (30)	g^+ 1^	alfalfa	90	-	plastic bag	-		bovine rumen	-	-	[35]

n.a.: not available due to limited access to the publication. n.r.: not reported. DM: dry matter. ^1^: scarified seeds. ^2^: ensiling durations; “n1–n2”: ensiling durations in the study differed for various reasons; “1, 2,…, n”: a specific time series of data was recorded.

#### 2.2.2. Data on Ensiling Treatments

The ensiling treatments in the studies differed in terms of ensiling duration, substrate, conditions, and silo scale (Table 1).

The duration of ensiling ranged from 1 to 281 days. For this review, ensiling durations from 1 to 30 days were classified as short-term (31% of all extracted data), from 31 to 90 days as medium-term (32%), and those lasting longer than 90 days as long-term ensiling (37%). Time series, i.e., sampling after different ensiling durations, were recorded in seven studies [11,13,21,28,29,33,34].

Seeds were ensiled in a variety of biomasses. Maize (*Zea mays*), alfalfa (*Medicago sativa*), and grasses were used most frequently. A comparison of ensiling seeds in different biomasses was carried out by Woodward [19], Anonymus [20], James et al. [32], Lück [22], Simard and Lambert-Beaudet [11], Piltz et al. [26], and Hahn et al. [12]. Variations in ensiled biomasses were also investigated, such as the comparison of chopped vs. unchopped biomasses [28]), the influence of additives (molasses [19], lactic acid bacteria [34], lactic acid [26], soil suspension [12]), and the effect of deliberately produced different DM or moisture contents [19,26,28,29].

For this review, the silo scale was classified as either lab-scale (e.g., plastic bags, glass jars, laboratory silos) or full-scale (e.g., on-farm silo bales and stacks). Of all data extracted, 54% were from lab-scale and 46% from full-scale silos. Two studies did not report this information or it was not available. Overud [28] and James et al. [32] compared the effects of different-sized silos on seed survival. The effect of the location (depth) of the seeds in the silo was investigated by Shevkenek [8], Tildesley [9], and James et al. [32].

In the repository the following parameters and variables for the ensiling treatments for each study were compiled, provided they were available: silo scale, silo type, ensiled substrates, information on chopping or wilting of the substrate, dry matter contents, pH values, any ensiling modifications, ensiling temperature and duration. As with the viability data, if more than one replicate was available, mean or median values were extracted.

#### 2.2.3. Data Analysis and Visualization

All statistical analyses on the extracted data were performed using the R environment version 4.4.0 [36]. Kruskal–Wallis rank sum tests were used to determine differences in survival rates between hardseeded and non-hardseeded species after ensiling; first for all data, then for data of surviving seeds only, and finally for all data at different ensiling durations. For data visualization, several R packages were used, namely, “ggplot2 version 3.5.1” [37], “dplyr version 1.1.4” [38], and “forcats version 1.0.0” [39]. The R package “webr version 0.1.5” [40] was used for the donut plots on seed survival and “vioplot version 0.5.0” [41] for the violin plots on changes in viability. Finally, the package “ggsankey version 0.0.99999” [42] was used for the Sankey diagram to visualize relationships between silo scale, plant family, and seed survival.

## 3. Key Findings on Seed Survival in Silages

### 3.1. Seeds Can Survive Ensiling

**Figure 1:**
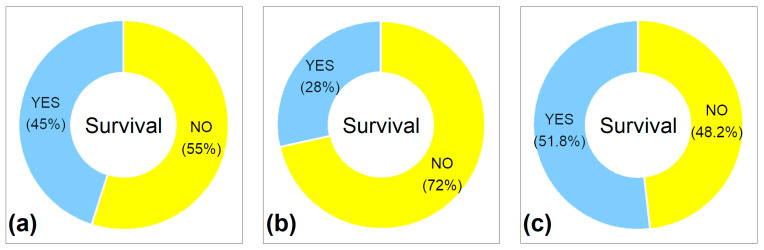
Overview of seed survival after ensiling, displayed for (**a**) the seeds of all 113 species tested (*n* = 424), (**b**) seeds of the 38 monocotyledonous species (*n* = 123), and (**c**) for the 75 dicotyledonous species (*n* = 301).

Ensiling could kill seeds but did not do so in every case. In fact, ensiling only completely killed the seed in 55% of all measurements reviewed. In 45% of the measurements, however, at least individual seeds were still viable (Figure 1a). Of the monocotyledonous species, 28% were still alive after ensiling, and 52% of the dicotyledonous species were alive (Figure 1b,c).

**Figure 2:**
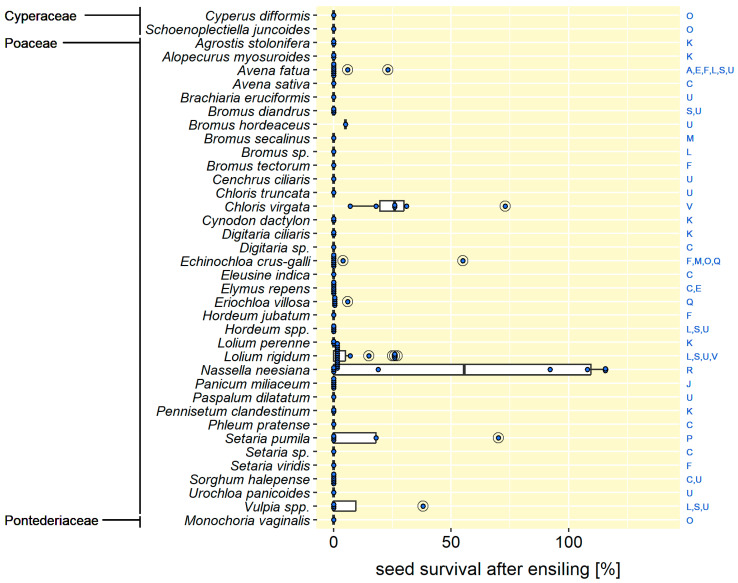
Survival probability of seeds of monocotyledonous species after ensiling. Blue dots and capital letters indicate the number of measurements and the examining studies for each species, respectively. Please refer to Table 1 for the assignment of the capital letters to the studies. Survival values above 100% imply an increase in observed viability due to ensiling.

**Figure 3:**
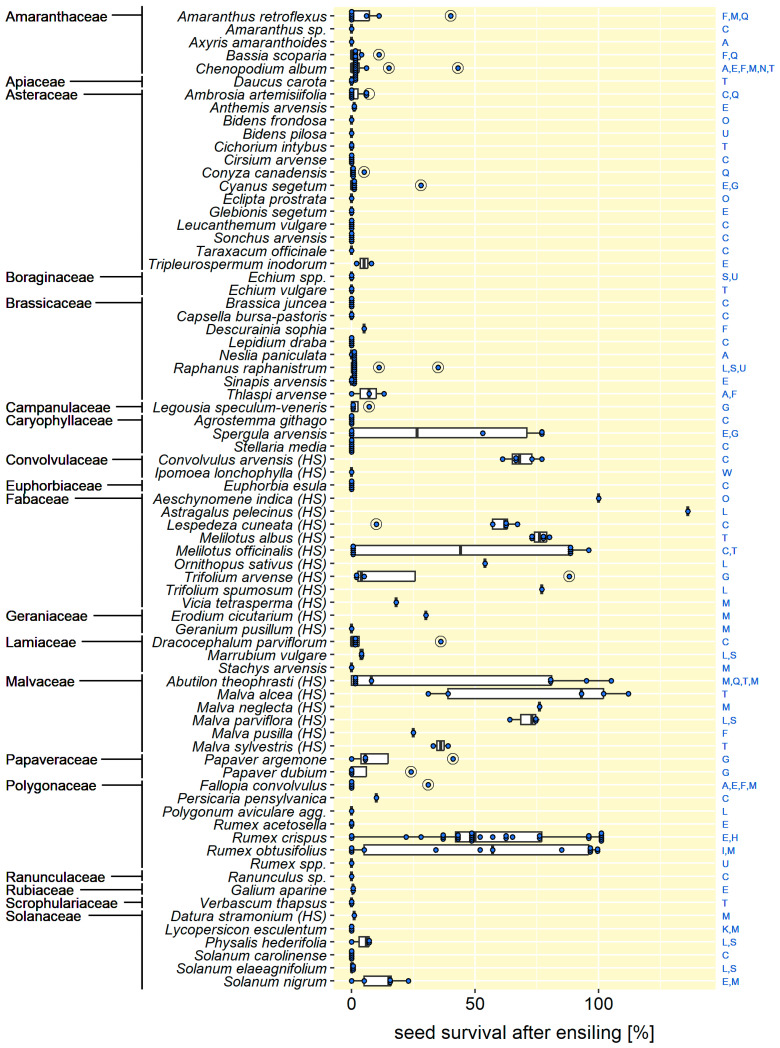
Survival probability of seeds of dicotyledonous species after ensiling. Blue dots and capital letters indicate the number of measurements and the examining studies for each species, respectively. Please refer to Table 1 for the assignment of the capital letters to the studies. Species with hardseededness are indicated by “(HS)”. Survival values above 100% imply an increase in observed viability due to ensiling.

In terms of species, 10 of the monocotyledonous and 43 of the dicotyledonous had the potential to survive ensiling. The monocotyledonous species, all of them Poaceae, capable of surviving ensiling treatments were as follows: *Avena fatua* (common wild oat), *Bromus hordeaceus* (soft brome), *Chloris virgata* (feathertop Rhodes grass), *Cynodon dactylon* (Bermuda grass), *Echinochloa crus-galli* (common barnyard grass), *Eriochloa villosa* (woolly cupgrass), *Lolium rigidum* (rigid ryegrass), *Nassella neesiana* (Chilean needlegrass), *Setaria pumila* (yellow bristle grass), and *Vulpia* spp. (silvergrass) (Figure 2). The following dicotyledonous species had the potential to survive ensiling: *Abutilon theophrasti* (velvet leaf, Malvaceae), *Aeschynomene indica* (Indian jointvetch, Fabaceae), *Amaranthus retroflexus* (red-root amaranth, Amaranthaceae), *Ambrosia artemisiifolia* (common ragweed, Asteraceae), *Anthemis arvensis* (field chamomille, Asteraceae), *Astragalus pelecinus* (biserrula, Fabaceae), *Bassia scoparia* (kochia, Amaranthaceae), *Chenopodium album* (common lambsquarters, Amaranthaceae), *Convolvulus arvensis* (field bindweed, Convolvulaceae), *Conyza canadensis* (Canada fleabane, Asteraceae), *Cyanus segetum* (cornflower, Asteraceae), *Datura stramonium* (thorn apple, Solanaceae), *Descurainia sophia* (flixweed, Brassicaceae), *Dracocephalum parviflorum* (dragonhead mint, Lamiaceae), *Erodium cicutarium* (common stork’s bill, Geraniaceae), *Fallopia convolvulus* (wild buckwheat, Polygonaceae), *Galium aparine* (cleavers, Rubiaceae), *Legousia speculum-veneris* (large Venus’s looking-glass, Campanulaceae), *Lespedeza cuneata* (Chinese bushclover, Fabaceae), *Malva alcea* (rose mallow, Malvaceae), *Malva neglecta* (dwarf mallow, Malvaceae), *Malva parviflora* (smallflower mallow, Malvaceae), *Malva pusilla* (round-leaved mallow, Malvaceae), *Malva sylvestris* (common mallow, Malvaceae), *Marrubium vulgare* (common horehound, Lamiaceae), *Melilotus albus* (white sweetclover, Fabaceae), *Melilotus officinalis* (yellow sweetclover, Fabaceae), *Ornithopus sativus* (Fench serradella, Fabaceae), *Papaver argemone* (pale poppy, Papaveraceae), *Papaver dubium* (long-headed poppy, Papaveraceae), *Persicaria pensylvanica* (Pennsylvania smartweed, Polygonaceae), *Physalis hederifolia* (ivyleaf groundcherry, Solanaceae), *Raphanus raphanistrum* (wild radish, Brassicaceae), *Rumex crispus* (curled dock, Polygonaceae), *Rumex obtusifolius* (broad-leaved dock, Polygonaceae), *Solanum elaeagnifolium* (silverleaf nightshade, Solanaceae), *Solanum nigrum* (black nightshade, Solanaceae), *Spergula arvensis* (corn spurry, Caryophyllaceae), *Thlaspi arvense* (field pennycress, Brassicaceae), *Trifolium arvense* (rabbitfoot clover, Fabaceae), *Trifolium spumosum* (bladder clover, Fabaceae), *Tripleurospermum inodorum* (scentless chamomille, Asteraceae), and *Vicia tetrasperma* (smooth vetch, Fabaceae) (Figure 3).

With regard to the above listing, it is important to note that the classification of a species as “capable to survive ensiling” cannot currently be applied generally but must be regarded as study-dependent. This is because of the fact that, first, the survival of most species was only tested in one study (82 species) or two studies (21 species). Only 10 of the 113 species were examined in at least three studies (Figure 2 and Figure 3). Second, because species tended to survive in one study but not in another. The four most frequently studied species illustrate this very clearly. These were the major weeds: *Avena fatua* [7,8,10,20,23,26], *Chenopodium album* [7,8,12,13,20,24], *Echinochloa crus-galli* [7,11,24,25], and *Fallopia convolvulus* [7,8,21,24]. The seeds of each of them survived in at least one study and were completely killed in at least one other study. Even within a single study, the conclusion on survivability could be contradictory, for example, due to different durations of ensiling (*C. album* in 13; *E. crus-galli* in 11) or different seed lots (*A. fatua* in 10).

### 3.2. Ensiling Reduced Seed Viability—In Most Cases

Viability after ensiling was usually reduced compared to untreated seed, even in 45% of cases where the seed survived. On average (median), the surviving seed retained only 27 ± 37% of its initial viability. For the surviving monocots, this proportion was 18 ± 35%; for the dicots, 35 ± 37% (Figure 2 and Figure 3).

Of the 53 species surviving ensiling, 35 retained not more than 10% of their initial viability (*n* = 75 measurements) and 8 species retained at least 75% (*n* = 42). These species have a high risk for surviving ensiling, namely, *Abutilon theophrasti*, *Aeschynomene indica*, *Astragalus pelecinus*, *Malva alcea*, *Melilotus officinalis*, *Nassella neesiana*, *Rumex crispus*, and *Rumex obtusifolius* (Figure 2 and Figure 3). The four important weeds mentioned above, *A. fatua*, *C. album*, *E. crus-galli*, and *F. convolvulus*, retained, on average (median of all studies), only 15 ± 50%, 15 ± 49%, 2 ± 49%, and 16 ± 50% of their initial viability after ensiling.

A special case was the species whose seeds (at least in some measurements) survived ensiling without any loss of viability. These were the monocot *Nassella neesiana* and the dicots *Abutilon theophrasti*, *Aeschynomene indica*, *Astragalus pelecinus*, *Malva alcea*, *Rumex crispus*, and *Rumex obtusifolius* (Figure 2 and Figure 3). Of these species, five were a particularly special case, as their observed seed viability after ensiling exceeded that in the untreated controls. This occurred in (mostly single) samples of *Abutilon theophrasti* [12], *Astragalus pelecinus* [23], *Malva alcea* [12], *Nassella neesiana* [34], and *Rumex crispus* [28]. In addition, it was also reported by Piltz et al. [26] that “the proportion of apparently viable non-germinated seeds appear[ed] to increase for some species [after ensiling treatments]” (p. 5). Unfortunately, the viability values were not reported. Nevertheless, these observations seem to indicate that ensiling can have not only an inactivating but also a viability-stimulating effect on seeds. This stimulation may, in some cases, e.g., in *N. neesiana*, be due to the breaking of dormancy and the initiation of germination by ensiling, as has also been observed by animal digestion or in biogas plants (e.g., [10,43,44]). However, in the case of *A. pelecinus* [23], *A. theophrasti* and *M. alcea* [12], and the investigations by Piltz et al. [26], not only germinability but also the metabolic activity of the ensiled seed increased. A similar increase of total viability was observed when seeds were exposed to anaerobic digestion in biogas reactors and linked to processes underlying hormetic responses (e.g., [45]). In the case of ensiling, there is a need for research into the triggering factors and the mechanisms behind the observed viability stimulation.

### 3.3. Some Seed Traits Promote Survival

**Figure 4:**
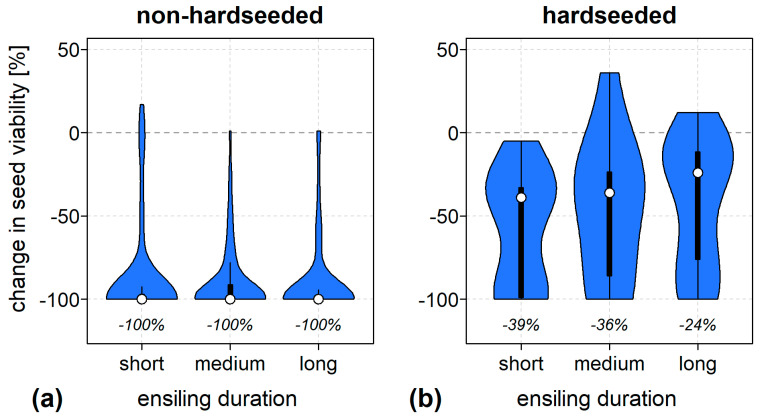
Changes in seed viability due to short- (1–30 days), medium- (31–90 days), and long-term (>90 days) ensiling of species (**a**) without and (**b**) with hardseeded seeds compared to untreated seed lots. Both germinated and metabolically active seeds (tetrazolium test) were considered viable. Sample sizes (number of species) were 113 (30), 124 (46), 128 (56), 17 (4), 11 (8), and 31 (12) for short-, medium-, and long-term ensiling of non-hardseeded (**a**) and hardseeded species (**b**), respectively. Values in italics below the violin plots are medians calculated from the extracted data summarized in the repository.

The survivability of seeds in silage varied depending on the species and study. Compared to untreated seeds, seed viability usually decreased in ensiling. However, the extent of this decrease differed not only between monocot and dicot species (see section above), but also within the dicots. For dicot species with the potential to form physically dormant, so-called hardseeded (HS), seeds appeared to be particularly resistant to ensiling (Figure 3 and Figure 4).

Seeds of HS species have one or more impermeable layers in the seed or fruit coat. Hardseededness is common in the family of Fabaceae but also occurs in members of Malvaceae, Geraniaceae, Convolvulaceae, and Solanaceae [46]. Ten of the reviewed ensiling studies examined the survival of a total of 20 HS species (Figure 3). Averaged over all measurements and ensiling durations (see Section 3.4), seeds of HS species lost significantly less viability than non-HS species, retaining a median of 64 ± 38% of its initial viability instead of being completely inactivated (*n* = 424, *p* < 0.001). The same applied when considering only the surviving species (*n* = 191, *p* < 0.001) or ensiling for short (*n* = 130), medium (*n* = 135), and long (*n* = 159) periods individually (*p* < 0.001 in each case) (Figure 4).

Eight species retained at least 90% of their initial viability after ensiling. Of these, five were HS, namely, *Abutilon theophrasti*, *Aeschynomene indica*, *Astragalus pelecinus*, *Malva alcea*, and *Melilotus officinalis* (Figure 3). However, three non-HS species, namely, the grass *Nassella neesiana* and two Polygonaceae *Rumex crispus* and *Rumex obtusifolius*, were also able to resist ensiling to this extent (Figure 2 and Figure 3). Consequently, there must be other, as yet unknown, seed traits that enable seeds to survive ensiling. Possibly the hardness (not hardseededness) or thickness of the seed coat plays a role here, both of which have been suggested to be relevant for the persistence of seeds in soil, animal digestive tracts biogas reactors (e.g., [47] and references therein). The seeds of *N. neesiana*, for example, are known to be hard and sharp and can be inadvertently dispersed with hay bales ([34] and references therein).

Further indications of seed traits favoring seed survival in ensiling could be obtained by comparing the survival of different seed lots of a single species examined in the same study. To date, data from five such studies are available. In the investigations by Blackshaw and Rode [7] and Overud [28], seed lots of the same species responded very similarly. Aper et al. [13], however, found that only two out of three tested *Chenopodium album* seed lots that were (not) resistant to different herbicides survived short-term ensiling in a silage stack. Piltz et al. [10] compared one- and two-year-old lots of eight species. In three species (*Avena fatua*, *Raphanus raphanistrum*, *Solanum elaeagnifolium*), the younger lot survived lab-scale ensiling for 90 days, and in one species (*Physalis hederifolia*), the older lot survived. Finally, Asaduzzaman et al. [27] reported differences in the survival probability of *Chloris virgata* lots of a different origin and/or age. Therefore, seed lot characteristics such as the degree of maturity appear to play a role in the survival of ensiling. This has also been found in studies investigating (in addition to ensiling) the survival of seeds in biogas reactors [7,10,13,24,27,44,45,48,49]. The effects of seed lot characteristics are likely to be particularly important for HS species. Variations in the degree and depth of HS may result in different survival rates, and these variations in HS can be caused by factors such as genetic differences, weather and site conditions, seed maturity, and endogenous dormancy rhythms [50,51,52,53]. Therefore, in the future, ideally, several seed lots of one species would be examined and the results obtained formulated with reference to these seed lots.

### 3.4. The Vague Role of Ensiling Conditions

Since the beginning of documented research on seed survival in silage, the question has arisen as to which factors affect seed viability. Already, Woodward [19] experimented with differing moisture contents and the addition of molasses. All studies—although in different resolutions—reported the duration of the ensiling treatments and the composition of the ensiled biomasses (Table 1). It can be assumed that these and other differences in the ensiling conditions had an influence on the survival of the seeds. Unfortunately, the silage substrates, their preparations, the ensiling environments, and the ensiling durations differed so greatly between the studies that a meaningful statistical meta-analysis was not possible. Nevertheless, trends could be identified from some studies.

It is important to bear in mind that two key factors of ensiling conditions are intertwined: the dry matter (DM) content of the substrate and the amount of organic acids formed in the feedstock. At substrate wilting levels of over 40% DM, the pH value of the silage can generally no longer be reduced to levels that cannot also occur in the soil, the natural environment of the seed. From this, it can be concluded that a reduction in seed survival at higher DM levels is more likely due to low concentrations of (dissociated) longer-chain fermentation acids and compounds such as esters and alcohols rather than lactic acid and total acidity, i.e., the low pH itself. However, these volatile compounds were rarely analyzed, and if so [12], then not in sufficient detail to link variations in seed viability to them.

Some authors have succeeded in reducing the survival rate of the seeds through the addition of molasses and lactic acid bacteria (e.g., [34]). Such additives can enhance lactic acid production and help to lower the pH value to a certain extent. Contrary to popular belief, however, the most striking effect of these additives is not the extent of the pH reduction, but the speed at which it occurs. As a result, the transition phase from the initial partially anaerobic to the strictly anaerobic phase of ensiling is shortened, and the effects of typical and important biochemical reductions, e.g., from nitrate and sulphate to nitrite and sulfide, are minimized. Therefore, silage additives tend to mask potential effects and can make it difficult to uncover the potential impact of the ensiling environment—beyond anoxia—on seed survival.

The aspect of chop-length was investigated and discussed by Piltz et al. [26] regarding its practical importance for short-chopped pile silos and longer chop-length in baled haylages. This differentiated view is very useful, and it is, therefore, worth reproducing such binary experimental approaches, especially when the surveys are conducted at the farm level. On a laboratory scale, it should be noted that short-chopped biomass is much easier to compact and the carbohydrate sources are more readily available for the lactic acid bacteria. This, in turn, will lead to faster acidification and, thus, to a similar effect to that achieved by the use of additives. Therefore, a direct, singularly responsible effect of chopping length cannot be recognized from the available studies and is also not to be expected. Rather, experimenters should be clear from the outset whether they want to simulate contemporary practical conditions in the best possible way or whether the processes of seed inactivation are the focus of interest. In the latter case, an intermediate chop-length would be sufficient.

The role of ensiling duration for seed survival could be assessed somewhat more clearly on the basis of the compiled results. In most cases, a longer duration of seeds in the silage feedstock was associated with a decrease in seed viability. Since this effect could be observed independently of the fermentation patterns, the pure exposure time to anoxia, modulated by the moisture content, was obviously the driving factor. This effect was most pronounced in seeds of dicotyledons with an intermediate survival rate (e.g., [29]), whereas the effect of silage exposure time could not be demonstrated for most of the soft-shelled gramineous seeds. The gramineous seed lots contained hardly any viable seeds after just a few days. Only in a few cases did an extension of the ensiling duration beyond 90 days lead to more differentiated findings on the survival rate of the ensiled seeds.

**Figure 5:**
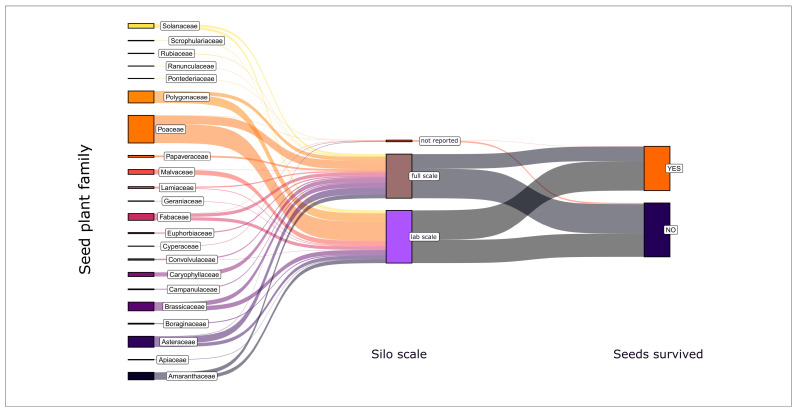
Sankey diagram illustrating the variation of seed survival of the tested plant families with respect to the scale of the silos (lab, full) in which they were incubated. The width of the flow lines corresponds to the number of studies.

Finally, it is worth taking a look at an ensiling condition that was reliably documented: the silo scale (Figure 5).

The survival of seeds from representatives of most plant families studied was tested both in full-scale and in lab-scale silos—albeit under different experimental circumstances by different authors. Whether the seed survived ensiling (YES/NO) was obviously barely influenced by the scale of the silo (Figure 5). The slightly higher proportion of dead seeds in the full-scale silos is probably more related to the higher proportion of Poaceae examined (and killed) in the full-scale silos compared to the lab-scale silos than to the difference in scale. From the almost congruent results on seed survival in full- and lab-scale silos (Figure 5), it may be concluded that it is not absolutely necessary to incubate a seed under practical conditions to determine its survival in ensiling. Consequently, for future studies aimed at clarifying the effects of ensiling conditions, lab-scale silos would be preferable to full-scale silos, as they are easier to control, standardize, and reproduce.

## 4. Implications for Sustainable Silage Production

Overall, the reviewed data sets on seeds in silage prove that seeds of some species have the potential to survive the ensiling process. According to the current state of knowledge, these mainly include HS species, but also the noxious grass *Nasella neesiana*. Thus, there is a risk that surviving seeds could be spread with the silage as residual biomass after feedstock utilization and cause weed problems. Increasing weed pressure is associated with additional costs and efforts to control it, which would have a negative effect on sustainability. Consequently, the aspect of seed survival should be taken into account in integrated sustainable silage production.

Including the survival of (weed) seeds in sustainability considerations is particularly important in view of the increasing diversification of the plant-biomass-based feedstocks used. On the one hand, this applies to the growing spectrum of species (e.g., [54,55,56]), among which are also seed-forming wild plants (e.g., [57]). On the other hand, this refers to the utilization of other, more diverse plant parts (keyword, whole plant silage) through which new, different, or more weed seeds could enter silage [4]. Most of the available studies today have focused on current weed and pasture species. The survival of the seeds of other species, however, is still largely unexplored, but see Hahn et al. [12] for an example. In addition, the production pathways involving ensiling are diversifying as well, e.g., as “cut and carry” biomasses in emerging stockless organic cropping systems [58]. The more diverse use of silage also entails new production conditions [59] that could influence the survival of the seed.

Apart from the risk that silage poses in terms of surviving (weed) seeds, silage production also offers an opportunity in this respect: Ensiling can reduce seed viability and, thus, contribute to weed control. However, it is currently not possible to quantify how effective this measure can be in the toolbox of integrated weed management. This is mainly due to the very heterogeneous database. Even after 90 years, the number of studies on the subject is limited and research approaches are very different. All 23 studies aimed to test whether seeds of the selected plant species could survive ensiling. The study designs and the degree of documentation, however, differed greatly with regard to the seed quantities examined, the type of seed response measured, and the ensiling conditions applied (see Table 1). This makes it difficult to draw generally applicable conclusions. For instance, the methods for determining seed survival differed in their informative value. While determining the germinability in pots provides information about seed vigor, germination on agar plates estimates the potential maximum germination capacity, and the metabolic activity can be used to assess the proportion of dormant seeds that potentially emerge. The use of different numbers of replicates, seeds per replicate, and various (uncharacterized) seed lots further complicates reaching robust conclusions on species likely to survive and factors driving seed inactivation in silage.

## 5. Emerging Fields of Research

In the interest of sustainable, weed-risk-free silage production, the primary goal of future research on seed survival in silage would be to determine which factors (combinations) contribute to seed killing and to be able to predict the survival probability of species that have not yet been tested. Considering the currently rather heterogeneous data situation, a systematic, extensively documented approach is recommended. This applies not only to the ensiling conditions, but also to the selection of species and seed lots. Methodologically, it would make sense to record time series during the ensiling process, model seed viability as a function of exposure time, and determine inactivation times. Additionally, comparisons could be made between silages that differ in individual factors such as dry matter content or fermentation patterns. Comparable studies from biogas reactors already exist (e.g., [17,45,60]). The knowledge gained in this way would be of interest for fundamental research in seed biology. For example, the factors and pathways by which ensiling inactivates seeds or inhibits/stimulates germination and metabolic activity require research. In addition, these findings could provide information for the optimization of silage production conditions with regard to weed seed management.

In addition to the question of whether and to what extent seeds can survive ensiling, the aspect of how the weed risk situation is to be assessed in practice must also be considered. It cannot be concluded from the possibility of their mere survival that there is a risk of contamination of silage by viable plant seeds. Thus, knowledge of the probability, the quality, and the quantity of seeds entering silage production is required. Influencing factors would be management parameters such as standing time, harvest time, and frequency as well as cutting height. In this context, it should be noted that the seeds used in the studies were usually dried and stored before the silage treatments, i.e., they were already mature. That means that their survivability probably differs from that of the freshly harvested seed that enters agricultural ensiling processes. Additionally, it should be borne in mind that the question of weediness of species also has a temporal component. Climate and land use changes may affect the establishment, control, and, ultimately, weed or invasive potential of plants [61,62,63]—including those that have the potential to survive ensiling.

Finally, several of the studies on seeds in silage have additionally investigated seed survival in treatments such as animal digestion and manure storage (Table 1). This reflects the fact that, in practice, entire agricultural process chains will determine whether entering seeds pose a weed risk. With regard to forage silage, animal mastication and digestion follow ensiling and the resulting manure is stored and probably digested. In biogas production, ensiled biomass and seeds undergo anaerobic digestion in biogas reactors and the storage of the digestate. It has already been demonstrated that seeds of some species have the potential to survive ensiling (this review), animal digestion (e.g., [7,64,65]), anaerobic digestion in biogas reactors [47], and storage of digestates (e.g., [66]). Even for the combination of some processes, the survival of some species has already been proven (e.g., [66]). However, seeds would have to survive the interaction of all these processes for the stored manure, digestate, or other resulting products to compromise the sustainability of closed material cycles. Whether or not seeds have the potential to do this needs to be determined.

## 6. Conclusions

The research for this review shows that the question of whether plant seeds can survive ensiling and, thus, become problematic from a weed control perspective has occupied researchers and practitioners for almost a century. Based on the data compiled, it is clear that some seeds can indeed survive ensiling, and consequently, an awareness of this issue is necessary for sustainable silage production. The current state of knowledge allows for a tendency-based assessment of the survival rate of a species in ensiling but no generally applicable prediction. Nevertheless, the comprehensive analysis and visualization of all available data in this review should allow farmers, researchers, and environmental authorities to form an impression and make decisions for or against the cultivation of species on this basis. Furthermore, it is clear that seed biology plays a crucial role in seed survival. Species with the ability to form physically dormant (hardseeded) seeds have a significantly higher risk of survival than other species. With regard to ensiling conditions, no individual factor(s) with a consistent effect on the seed survival of all species could be identified. It seems that the effects of the ensiling conditions are very case-specific and the interactions with the silo microbiome are extremely diverse. Consequently, the question of seed survival in silage opens up a wide field for future research into key areas such as seed biology, ensiling conditions, weed spread, and the selection of plant species suitable for sustainable silage production.

## Data Availability

The original data presented in the study are openly available at https://zenodo.org/records/14629213 (accessed on 13 January 2025).
